# Selection of suitable biomass conservation process techniques: a versatile approach to normal wiggly interval-valued hesitant fuzzy set using multi-criteria decision making

**DOI:** 10.1007/s40747-023-01097-1

**Published:** 2023-05-29

**Authors:** Samayan Narayanamoorthy, L. Ramya, Angappa Gunasekaran, Samayan Kalaiselvan, Daekook Kang

**Affiliations:** 1 Department of Mathematics, Bharathiar University, Coimbatore, 641046 India; 2 School of Business and Public Administration, California State University, Bakersfield, 9001, Stockdale Highway, 20BDC/140, Bakersfield, CA 93311-1022 USA; 3 Department of Social Work, SRMV Collge of Arts and Science, Coimbatore, 541020 India; 4 Department of Industrial and Management Engineering, Inje University, 197 Inje-ro, Gimhae-si, Gyeongsangnam-do Republic of Korea

**Keywords:** NWHFs, IVHFs, Biomass conversion processes, DEMATEL, PROMETHEE

## Abstract

A country that relies on developing industrialization and GDP requires a lot of energy. Biomass is emerging as one of the possible renewable energy resources that may be used to generate energy. Through the proper channels, such as chemical, biochemical, and thermochemical processes, it can be turned into electricity. In the context of India, the potential sources of biomass can be broken down into agricultural waste, tanning waste, sewage, vegetable waste, food, meat waste, and liquor waste. Each form of biomass energy so extracted has advantages and downsides, so determining which one is best is crucial to reaping the most benefits. The selection of biomass conversion methods is especially significant since it requires a careful study of multiple factors, which can be aided by fuzzy multi-criteria decision-making (MCDM) models. This paper proposes the normal wiggly interval-valued hesitant fuzzy-based decision-making trial and evaluation laboratory model (DEMATEL) and the Preference Ranking Organization METHod for Enrichment of Evaluations II (PROMETHEE) for assessing the problem of determining a workable biomass production technique. The proposed framework is used to assess the production processes under consideration based on parameters such as fuel cost, technical cost, environmental safety, and $CO_2$ emission levels. Bioethanol has been developed as a viable industrial option due to its low carbon footprint and environmental viability. Furthermore, the superiority of the suggested model is demonstrated by comparing the results to other current methodologies. According to comparative study, the suggested framework might be developed to handle complex scenarios with many variables.

## Introduction

Increasing demand for energy, the gradual depletion of renewable energy and the environmental problems caused by the consumption of renewable energy are the challenges that all countries must face when dealing with the relationship between energy, economy and environment. Biomass can make a substantial contribution to supplying future energy demand in a sustainable way. Biomass energy is solar energy stored in the chemical bonds of carbon and hydrogen chains as a result of photosynthesis or the metabolic activity of organisms. It is presently the largest global contributor of renewable energy, and has significant potential to expand in the production of heat, electricity, and fuels for transport. At present, forestry, agricultural and municipal residues, and wastes are the main feedstocks for the generation of electricity and heat from biomass. In addition, a very small share of sugar, grain, and vegetable oil crops are used as feedstocks for the production of liquid biofuels. The nomenclature is presented in Table 1.

**Table 1:** Nomenclature

MCDM	Multi-criteria decision making
MAGDM	Multi-attribute group decision making
HFS	Hesitant fuzzy set
IVHFS	Interval-valued hesitant fuzzy set
NWHFs	Normal wiggly hesitant fuzzy set
NWIVHFS	Normal wiggly interval-valued hesitant fuzzy set
NWIVHFN	Normal wiggly interval-valued hesitant fuzzy number
AHP	Analytic hierarchy process
TOPSIS	Technique for order preference by similarity to ideal solution
VIKOR	VlseKriterijuska Optimizacija I Komoromisno Resenje
DEMATEL	Decision-making trial and evaluation laboratory model
PROMETHEE II	Preference Ranking Organization METHod for Enrichment of Evaluations II
ELECTRE	Elimination Et ChoixTraduisant la REalite
WPM	Weighted product method
WSM	Weighted sum method

Today, biomass supplies some 50 EJ [4] globally, which represents 10% of global annual primary energy consumption. The production of heat by the direct combustion of biomass is the leading bioenergy application throughout the world, and is often cost competitive with fossil fuel alternatives. For a more energy efficient use of the biomass resource, modern, large-scale heat applications are often combined with electricity production in combined heat and power (CHP) systems. Each bioenergy technology has its own technical challenges to overcome that depend mostly on their technical, environmental, and social status. The evaluation of multiple energy options is often complicated, and it is difficult to maximize all decision criteria when selecting alternatives. With the expansion of the standards scope, there may be interactions and conflicts between them. Therefore, decision makers need to make some compromises among different standards. In this context, multi-criteria decision-making (MCDM) methods are gaining more and more attention. An improved normal wiggly interval-valued hesitant fuzzy (NWIVHF), decision-making trial and evaluation laboratory (DEMATEL)-Preference Ranking Organization Methods for Enrichment Evaluation (PROMETHEE) method is proposed to rank alternatives and characterize certain factors.

The purpose of this study is to provide a comprehensive evaluation decision-making framework to develop the renewable energy processing techniques. Firstly, the evaluation criteria, energy situation, and renewable energy are determined from the perspectives of energy, economy, technology, and environment, and establishing the energy evaluation processes for sustainable development. Then the sustainable development comprehensive processes of each alternative is calculated by MCDM methods, and the best renewable energy scheme is ranked.

The remainder of this article is structured as follows. In “Literature review”, we review and summarize the relevant literature; in “Preliminaries”, the method description and data sources are given; in “Methodology and problem formulation”, the results are discussed; in “Illustrative example”, conclusions and recommendations are discussed.

## Literature review

MCDM is essential for choosing a nation’s sustainable energy source. Researchers from several fields have recently started using MCDM technology to find clean energy alternatives for problems relating to energy. Here, we outline the numerous studies that have been done in relation to biomass energy, NWIVHFS, DEMATEL, and PROMETHEE-II methods.

Alsaleh et al. [5] recognized the consequences for the EU28 area of the internal (region-specific) and external (economic and financial) predictors of the level of intellectual productivity in the bioenergy sector. Ballarin et al. [6] set out to determine how cultivation methods may improve cellulosic ethanol production. Gitinavard et al. [14] proposed a new decision-making methodology that is implemented based on a novel equilibrium ranking system and elapsed time-valued hesitant fuzzy sets for energy judgment-making problems through multiple parameters. Khan [17] analyzed the internal and external climate of the CNG industry in Iran using SWOT (strengths, weaknesses, opportunities, and threats) analysis to prioritize strategies for stimulation of the economy of the Iranian CNG market. Global bioenergy innovations were designed, characterized, and selected in accordance with 15 criteria for biodiversity by Khishtandar et al. [20]. Van de Kaa et al. [42] investigated the Dutch battle against fuel conversion innovation. Their findings imply that gasification by deforestation has the greatest potential for standard supremacy.

Kheybari et al. [19] classified biofuel processing technologies using the AHP approach. Cutz et al. [12] used the fuzzy MCDM approach to define a portfolio of CA-appropriate technologies for biomass conversion, taking into account technological, cultural, ecological, as well as economic–political aspects. Lerche et al. [22] employed the MCDM process to evaluate renewable bioenergy sources in small towns and villages in the Netherlands and in Saxony, Germany. Rodrguez et al. [38] enhanced the fuzzy AHP integrated GIS formulation, which includes both geographical and quasi-spatial variables for determining the best location for a bioenergy facility. Supriya et al. [40] provided a review that combined federated learning systems with soft computing methodologies. Ramesh et al. [36] developed a hybrid MCDM framework for prioritizing the lignocellulose biomass for the production of bioethanol as an alternative fuel for automobiles. For selecting the appropriate biomass material for biofuel production, Firouzi et al. [13] employed the hybrid TOPSIS–ARAS–WASPAS technique. Ossei-Bremang et al. [34] suggested a triangular fuzzy-based decision model to assess the different biomass resources for bioenergy production. Yenduri et al. [43] used the TOPSIS technique to analyze the software maintainability prediction models. Ilbahar et al. [15] developed a Pythagorean fuzzy-based MCDM framework for analyzing the biomass conversion technologies for Turkey and combustion was obtained as the feasible conversion technology. Khadivi et al. [18] discussed the investment plans for the production of syngas and renewable natural gas through biomass gasification based on MCDM. Bisht et al. [8] developed an integrated Delphi-AHP framework to assess the optimal plant size selection for biomass gasification based on the technical, economic, social, and environmental aspect.

Abdullah et al. [1] recommended interval-valued intutionistic fuzzy DEMATEL combined with the choquet integral for evaluation of solid waste management problem. Asan et al. [3] proposed an significant inconsistencies and observable behaviors are inherent in the decision process. They declare a new interval-assessed, reluctant, fuzzy model for DEMATEL that has the skills to deal directly with expert appraisal reluctance and provide a fair representation of ambiguity. Baykasoğlu et al. [7] proposed an integrted fuzzy DEMATEL–TOPSIS framework to model and solve a land transport company’s problem of transport distribution. Liu et al. [25] introduced an integration structure of the IVHF–DEMATEL system improved by the AVL (average vector length) operator on CVPs. Geetha et al. [39] evaluated the various renewable energy sources involving the hesitant Pythagorean fuzzy DEMATEL–VIKOR methods. Narayanamoorthy et al. [31] evaluated the alternative fuel selection problem involving the DEMATEL–COPRAS approach. Jiang et al. [16] analyzed the factors hindering the sustainable development in biomass in Tehran using the interval-valued spherical fuzzy BWM–DEMATEL methods.

Chen [10] established a type-2 fuzzy PROMETHEE-II framework that uses an expected value-based overriding correlation methodology within the Type-2 fuzzy variable sets ecosystem. Liang et al. [24] used a hesitant fuzzy linguistic fuzzy term set-based PROMETHEE technique for evaluating green environmental management. A selection of feasible biomass material for maximum yield of bio-oil during pyrolysis have been carried out using the PROMETHEE-II method by Madhu et al. [29]. Chen et al. [11] proposed a picture linguistic fuzzy-based PROMETHEE-II method for assessing the renewable source for China. Narayanamoorthy et al. [32] used the intuitionistic fuzzy soft PROMETHEE-II approach to assess the preference for the COVID-19 vaccination. Li et al. [23] evaluated the various renewable energy options suitable for the western Chinese city using the probabilistic linguistic fuzzy DEMATEL–PROMETHEE method. Agrawal [2] evaluated the supplier selection problem based on the PROMETHEE-II method. The application of NWHFS in capturing uncertainity in dealing with different MCDM problems are presented in Table 2.

**Table 2:** NWHFS in MCDM approaches

Authors	Proposed approach	Implementation
Ramya et al. [37]	NWIVHF Pythagorean-based SWARA–WASPAS technique	Selection of thermal energy storage technique
Ren et al. [35]	NWHFS	Environmental quality evaluation problem
Narayanamoorthy et al. [30]	NWHF VIKOR method	Site selection for hydrogen underground storage
Liu et al. [26]	NWHF linguistic MABAC approach	Evaluation of marine ecological security situation
Liu et al. [27]	NWHF MABAC–CCSD method	College book supplier selection problem
Liu et al. [28]	NWHF TODIM approach	High-level personnel training evaluation problem
Zhang et al. [45]	NWHF CCSD–WASPAS	Electric vehicle charging station selection

### Main motivation and contribution of this research

The main purpose of this research was to select the best biomass conservation process techniques. The alternative that was selected was classified and selected based on the characteristics of the selected criteria. The benefit of choice is protection of the renewable energy source in a manner that is not harmful to the environment and is beneficial to humans. Here, the NWIVHF set, an extended set of the NWHF set, is proposed. The NWHF set provides value-added solutions to deep and deeper ideas in the mind of the decision maker. The solution with the NWHF set is a decisive and clear solution. The NWHF set with such features extends to the NWIVHF set.

The NWHFS, HFS, IVHFS, and PHFS each have their own unique characteristics. NWIVHFS offers a clear and unbiased solution that combines membership and non-membership values with upper limit lower, limit values. In MCDM methods, NWIVHFS will correct the decision maker’s hesitation and perform various tests and provide solutions. In addition, for the NWIVHFS, two special methods, DEMATEL and PROMETHEE, have been proposed for incorporation into the MCDM method. The NWIVHF-DEMATEL method is used to calculate the importance of the criteria. The NWIVHF-DEMATEL weight detection method is based on the cause and effect relationships between the criteria.

NWIVHF-PROMETHEE method is used to rank the alternatives. The NWIVHF- PROMETHEE ranking method is based on leaving flows and entering flows; and these two are based on the net flows values.

## Preliminaries

### Definition 3.1

Let *U* be a finite hesitant fuzzy reference set of [0, 1]. The form of hesitant fuzzy set is as follows [41]:

$$\begin{aligned} B=\{ < u,\upsilon _{H}(u)> / u \in U\}, \end{aligned}$$

where $\upsilon _{H}(u)$ is the set of numbers from [0, 1]. The possible membership degrees of the element $u \in U$ to the set H and $\upsilon _{H}(u)$ are called the hesitant fuzzy elements.

### Definition 3.2

(IVFS) Let *U* be a fixed set. An IVFS *S* on *U* is described as follows [25]:

1 $$\begin{aligned} S=\left( \left\langle u, \lambda _H (u)\right\rangle / u \in U\right) . \end{aligned}$$

Here, $\lambda _H (u) \epsilon [0,1].$ Here, the intervals represent the possible membership degree. The pair of IVFN is written as $\left[ \lambda _H ^L (u), \lambda _H ^U (u) \right] .$

### Definition 3.3

Let *U* be a fixed set. An IVHFS $\tilde{p}$ on *U* is explained as follows [25]:

2 $$\begin{aligned} \tilde{A}=\left\{ \left\langle u_i (\tilde{\upsilon }_{\tilde{A}}(u_i)) / u_i \in U \right\rangle \right\} . \end{aligned}$$

Here, the set of interval is $\tilde{\upsilon }_{\tilde{A}}(u_i)$ in [0, 1]. These intervals represent the possible membership degree. $\forall u_i \in U,$ where $\tilde{A}= \left( \tilde{\upsilon }_{\tilde{A}}(u_i)\right)$ is the interval-valued hesitant fuzzy element.

$$\begin{aligned} \left( \tilde{\upsilon }_{\tilde{A}}(u_i)\right) = \left\{ \tilde{\psi } / \tilde{\psi } \in \tilde{\upsilon }_{\tilde{A}}(u_i) \right\} . \end{aligned}$$

Here,

$$\begin{aligned} \tilde{\psi }= \left( \left[ \lambda _H ^L (u), \lambda _H ^U (u) \right] \right) \end{aligned}$$

are called intervals numbers.

### Definition 3.4

Let $\tilde{A}=\left\{ \upsilon _{1},\upsilon _{2} \right\}$ be the reference set, and IVHFES, $\mu _{\tilde{A}}(u_{1})=\left\{ [0.2, 0.4], [0.3, 0.6]\right\}$ and $\mu _{\tilde{A}}(u_{2})=\left\{ [0.2, 0.3], [0.4, 0.5], [0.6, 0.7]\right\}$, its represents possible membership degree of $\mu _{i}(i=1,2)$ to the set *A*,  respectively, here, $\tilde{A}$ as IVHFS,

$$\begin{aligned} \tilde{A}= & {} \left\{ \left\langle \mu _{1},\left\{ [0.2, 0.4],[0.3, 0.6]\right\} \right\rangle ,\right. \\{} & {} \left. \left\langle \mu _{2}, \left\{ [0.2, 0.3],[0.4, 0.5], [0.6, 0.7]\right\} \right\rangle \right\} . \end{aligned}$$

## Methodology and problem formulation

###  Normal wiggly interval-valued hesitant fuzzy set

#### Definition 4.1

Let $U=\left\{ (u, h(u))/ u \in U\right\}$ be the hesitant fuzzy set (HFS) and the reference set of *U*. Then, NWHFS is represented as follows [35]:

3 $$\begin{aligned} NW=\left\{ \left\langle u, h(u), \phi (h(u))\right\rangle / u \in U\right\} . \end{aligned}$$

Here, *h*(*u*) represents the hesitant fuzzy element (HFE). Then, $\phi (h(u))$ represents the normal wiggly hesitant fuzzy element.

#### Definition 4.2

Let $NW=\left\{ \left\langle u, h(u), \phi (h(u))\right\rangle / u \in U\right\}$ be a reference set of *U*. The NWIVHFS on *U* can be defined as follows:

4 $$\begin{aligned} U_{NWIVHFS}=\left\{ \left\langle u_{i}, \tilde{\upsilon }_{\tilde{U}}(u_{i}), \tilde{\alpha }(\tilde{\upsilon }_{\tilde{U}}(u_{i}))\right\rangle u\in U\right\} . \end{aligned}$$

Here, $\tilde{\alpha }(\tilde{\upsilon }_{\tilde{U}}(u_{i})):U\rightarrow [0, 1]$ represents the possible membership degree of normal wiggly interval-valued hesitant fuzzy set. Each element of $u_{i}\in U$. The normal wiggly interval-valued hesitant fuzzy set element $\tilde{\upsilon }_{\tilde{U}}(u_{i})$ is denoted as

$$\begin{aligned} \tilde{\upsilon }_{\tilde{U}}(u_{i})=\left\{ \tilde{\delta }|\tilde{\delta }\in \tilde{\upsilon }_{\tilde{U}}(u_{i})\right\} . \end{aligned}$$

In the above equation, $\tilde{\delta }=[\tilde{\delta }^{L}, \tilde{\delta }^{U}]$ is the interval number of the upper and lower value. The lower and upper limits are denoted as $\tilde{\delta }^{L}=inf \tilde{\delta }$ and $\tilde{\delta }^{U}=sup \tilde{\delta }$.

$$\begin{aligned} \alpha (\tilde{\upsilon }_{\tilde{U}}(u_i))= & {} \left\{ [\delta _1^L,\delta _1^U],[\delta _2^L,\delta _2^U]... [\delta _n^L,\delta _n^U] \right\} \\= & {} \left\{ max (\delta _{i}^L {-} \tilde{f} (\delta _{i}^L)-0), ( 2rpd (\tilde{\delta }_{\tilde{U}}^L (u_i))\right. \\{} & {} \quad \left. {-}1) \tilde{f} (\delta _{i}^L){+} \delta _{i}^ L, min (\delta _{i}^ L{+} \tilde{f} (\delta _{i}^L),1 ) \right\} , \\= & {} \left\{ max (\delta _{i}^U {-} \tilde{f} (\delta _{i}^U)-0), ( 2rpd (\tilde{\delta }_{\tilde{U}}^U (u_i))\right. \\{} & {} \quad \left. {-}1) \tilde{f} (\delta _{i}^U) {+} \delta _{i}^ U, min (\delta _{i}^ U{+} \tilde{f} (\delta _{i}^U),1 ) \right\} , \end{aligned}$$

where $$ u_{i}$$ is the value of $$ \tilde{\upsilon }_{\tilde{U}}(u_i) $$.

#### Definition 4.3

Let $\left\{ \left\langle u_{i}, \tilde{\upsilon }_{\tilde{U}}(u_{i}), \tilde{\alpha }(\tilde{\upsilon }_{\tilde{U}}(u_{i}))\right\rangle \right\}$ be NWIVHFE, then, the score function $\left\langle u, \tilde{\upsilon }_{\tilde{U}}(u_{i}), \tilde{\alpha }(\tilde{\upsilon }_{\tilde{U}}(u_{i}))\right\rangle$ follows as:

5 $$\begin{aligned}{} & {} \!\!\!S_{NWIVHPFS} \nonumber \\{} & {} \qquad = \frac{1}{2}\Bigg [\Bigg ( \mu (\bar{\upsilon }^{L}- \sigma _{\upsilon }^{L}) + (1-\mu ) \Bigg (\frac{1}{\# \upsilon } \sum _{i=1}^{\# \upsilon } \tilde{\bar{\upsilon _i}}^{L}-\sigma _{\tilde{\delta _i}}^{L}\Bigg ) \Bigg )^{2} \nonumber \\{} & {} \qquad +\Bigg ( \mu (\bar{\upsilon }^{U}- \sigma _{\delta }^{U}) + (1-\mu ) \Bigg ( \frac{1}{\# \upsilon } \sum _{i=1}^{\# \upsilon } \tilde{\bar{\upsilon _i}}^{U} -\sigma _{\tilde{\delta _i}}^{U}\Bigg ) \Bigg )^{2} \Bigg ],\nonumber \\ \end{aligned}$$

where

$$\begin{aligned} \bar{{\tilde{\upsilon }}}_{i} = \frac{\left( [\upsilon _{i}^{L}, \upsilon _{i}^{U}]\right) ^{L} + \left( [\upsilon _{i}^{L}, \upsilon _{i}^{U}]\right) ^{M} + \left( [\upsilon _{i}^{L}, \upsilon _{i}^{U}]\right) ^{U} }{3} \end{aligned}$$

and

$$\begin{aligned} \sigma _{\tilde{\upsilon _i}}=\sqrt{ \left( [\upsilon _{i}^{L}, \upsilon _{i}^{U}]^{L} \right) ^{2} {+} \left( [\upsilon _{i}^{L}, \upsilon _{i}^{U}]^{M} \right) ^{2} {+} \left( [\upsilon _{i}^{L}, \upsilon _{i}^{U}]^{U} \right) ^{2} {-} \left[ (\upsilon _{i}^{L}, \upsilon _{i}^{U} )^{L} (\upsilon _{i}^{L}, \upsilon _{i}^{U} )^{M} \right] } \\ {-} {\left[ (\upsilon _{i}^{L}, \upsilon _{i}^{U} )^{L} (\upsilon _{i}^{L}, \upsilon _{i}^{U} )^{U} \right] {-} \left[ (\upsilon _{i}^{L}, \upsilon _{i}^{U} )^{M} (\upsilon _{i}^{L}, \upsilon _{i}^{U} )^{U} \right] }. \end{aligned}$$

Here, $$\upsilon \in [0, 1].$$

#### Definition 4.4

An NWTIVHFs, $\tilde{c}$ on *U* is in terms of function $F_{\tilde{c}}(u).$ It defines a different NWTIVHFs.

6 $$\begin{aligned} \tilde{c}_{NWIVHF} = \left\{ \left\langle u,F_{\tilde{c}}(u)\right\rangle / u \in U\right\} . \end{aligned}$$

Here, $F_{\tilde{c}}(u)$ represents the IVTHFE and also provides some numerable NWIVTHFN.

7 $$\begin{aligned} F_{\tilde{c}}(u)= & {} \{ \upsilon _i\} \nonumber \\= & {} \left\{ p \left( [\upsilon _{i}^{L}, \upsilon _{i}^{U}]^{L}, [\upsilon _{i}^{L}, \upsilon _{i}^{U}]^{M}, [\upsilon _{i}^{L}, \upsilon _{i}^{U}]^{U} \right) / p(\upsilon _i) \right. \nonumber \\{} & {} \left. \in F_{\tilde{c}}(u), i=1,2,... \#F_{\tilde{c}}(u) \right\} , \end{aligned}$$

where $(\upsilon _{i})$ represents the triangular NWIVHFs.

Here, $\left( [ \upsilon _{i}^{L}, \upsilon _{i}^{U}]^{L} \le [\upsilon _{i}^{L}, \upsilon _{i}^{U}]^{M} \le [\upsilon _{i}^{L}, \upsilon _{i}^{U}]^{U} \right)$ denotes the triangular lower, middle and upper value and $[ \upsilon _{i}^{L}, \upsilon _{i}^{U}]$ represents the lower and upper limit of the NWIVTHFs. Then, $\# F_{\tilde{c}}(u)$ is the number of NWIVHFNs. Here, $\alpha ( \tilde{\upsilon }_{\tilde{p}} (u_i)): U \rightarrow [0,1]$.

### Proposed normal wiggly interval-valued hesitant fuzzy DEMATEL method (NWIVHF-DEMATEL)

**Fig. 1:**
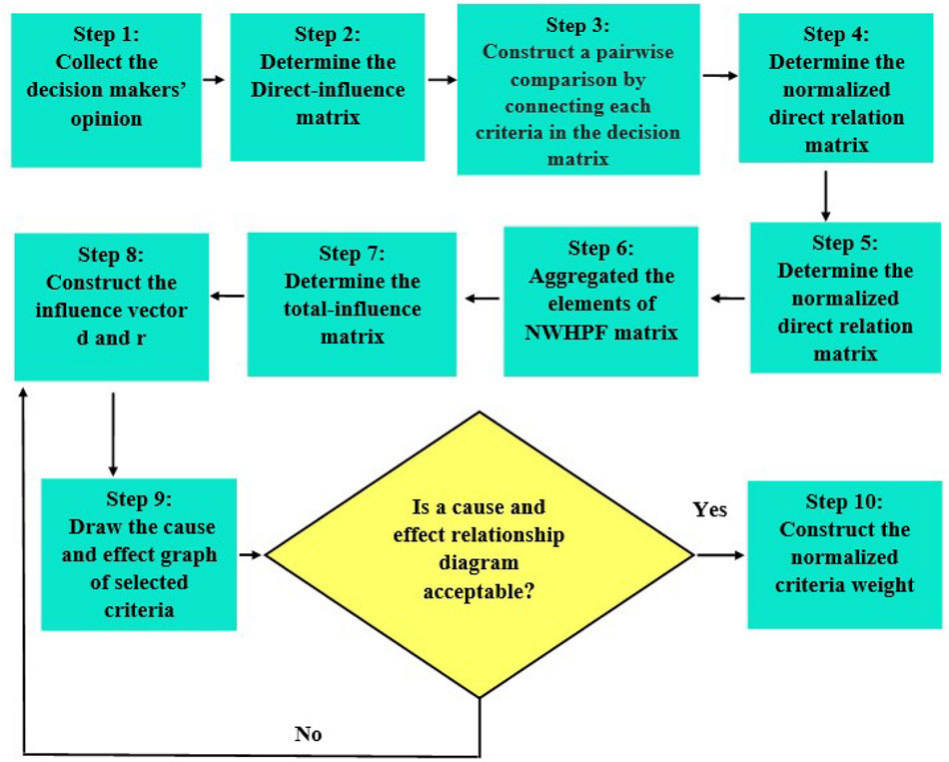
Flowchart of the NWIVHF-DEMATEL procedure

In this subsection, we propose a MCDM method called normal wiggly interval-valued hesitant fuzzy DEMATEL. Figure 1 shows the procedure of the NWIVHF-DEMATEL method.

There are eight steps in the algorithm, as follows.

**Step 1:**

**Fig. 2:**
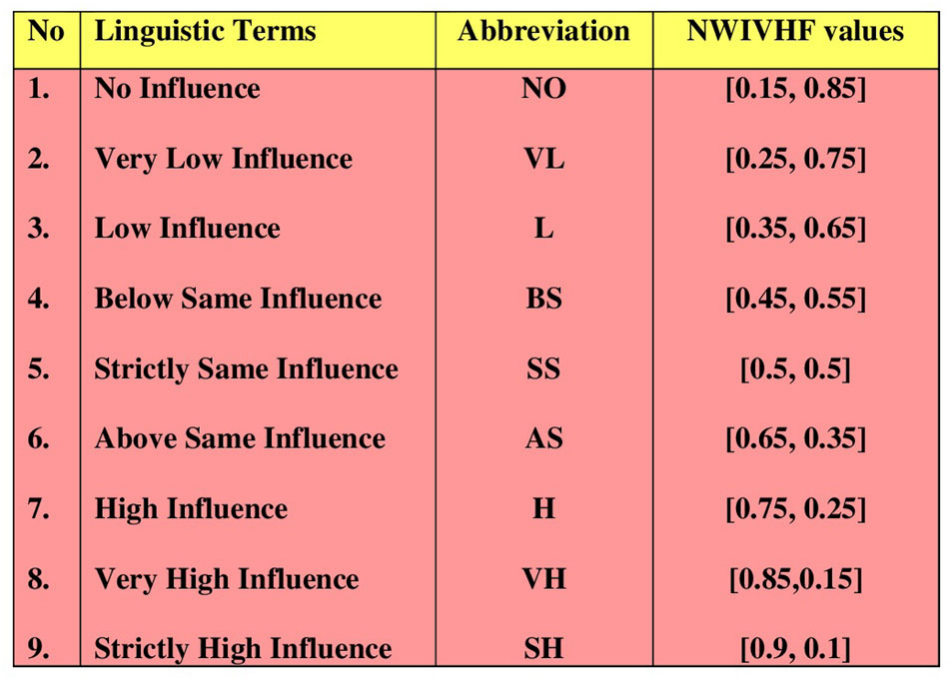
Linguistic scale for NWIVHF-DEMATEL

Create a direct-influence matrix. To that end, we assign the binding in the criterion structure. Decision makers determine each criterion value. Figure 2 shows the linguistic scale.

**Step 2:**

We create a direct-influence matrix. The value of each criterion is defined as *i*,  and each of the other criteria is defined as *j* It is defined by decision makers based on the following form:

8 $$\begin{aligned}{} & {} Z_{ij}=\{{\upsilon _{ij}^n}\} \nonumber \\{} & {} Z_{ij}=\{({\upsilon _{ij}^1}, {\upsilon _{ij}^2},...,{\upsilon _{ij}^n})\}. \end{aligned}$$

**Step 3:**

Pairwise comparison of bonding is developed by decision makers to each criterion based on the decision matrix as given below:

$$\begin{aligned}D=\begin{bmatrix} 0 &{}\quad d_{12} &{}\quad d_{13} &{}\quad \ldots &{}\quad d_{1n}\\ d_{21} &{}\quad 0 &{}\quad d_{23} &{}\quad \ldots &{}\quad d_{2n}\\ \vdots &{}\quad \vdots &{}\quad \vdots &{}\quad \ddots &{}\quad \vdots \\ d_{n1} &{}\quad d_{n2}&{}\quad d_{n3} &{}\quad \ldots &{}\quad 0 \end{bmatrix}.\end{aligned}$$

Experts analyze whether there is a relationship between the criteria or not. The membership degree assigns the subintervals [0, 1]. The NWIVHFE matrix can be represented as follows. The subintervals are arranged in the form of $[\tilde{\upsilon }^{L}_{ij}, \tilde{\upsilon }^{U}_{ij} ]$. In this form *i*, *j* represent the row and column values. These intervals are called the influence factors of possible membership degrees.

$$\begin{aligned}D=\begin{bmatrix} 0\quad &{}\quad {\upsilon _{12}^n} &{}\quad {\upsilon _{13}^n}&{}\quad \ldots &{}\quad {\upsilon _{1n}^n}\\ {\upsilon _{21}^n}&{}\quad 0 &{}\quad {\upsilon _{23}^n} &{}\quad \ldots &{}\quad {\upsilon _{2n}^n}\\ \vdots &{}\quad \vdots &{}\quad \vdots &{}\quad \ddots &{}\quad \vdots \\ {\upsilon _{n1}^n} &{}\quad {\upsilon _{n2}^n}&{} \quad {\upsilon _{n3}^n}&{}\quad \ldots &{}\quad 0 \end{bmatrix}.\end{aligned}$$

**Step 4:**

The following equation is used to construct the normalized direct relation matrix.

9 $$\begin{aligned}{} & {} \upsilon ^{L}=\zeta {\upsilon _{n1}^{L+}}+(1-\zeta ){\upsilon _{n1}^{L-}},\end{aligned}$$

10 $$\begin{aligned}{} & {} \upsilon ^{U}=\zeta {\upsilon _{n1}^{U+}}+(1-\zeta ){\upsilon _{n1}^{U-}}, \end{aligned}$$

where

$$\begin{aligned}{} & {} {\upsilon _{n1}^{L+}}= maximum \ \ {\upsilon ^{L}} \, \ \ \ \ {\upsilon _{n1}^{L-}}= minimum \ \ {\upsilon ^{L}}, \\{} & {} {\upsilon _{n1}^{U-}}= maximum \ \ {\upsilon ^{U}} \, \ \ \ \ {\upsilon _{n1}^{U-}}= minimum \ \ {\upsilon ^{U}}. \end{aligned}$$

Here, risk preference is denoted by $\zeta$, then the lower and upper limit values demote $\upsilon ^{L}$, $\upsilon ^{U}$. These $\upsilon ^{L}$ and $\upsilon ^{U}$ are based on the risk preference ($\zeta$) value, which is 0.5. The rewritten direct-influence matrix depends on the formula:

$$\begin{aligned} \tilde{D}=\begin{bmatrix} 0 &{}\quad ({\tilde{\upsilon }_{12}^L}, {\tilde{\upsilon }_{12}^U}) &{}\quad ({\tilde{\upsilon }_{12}^L}, {\tilde{\upsilon }_{12}^U}) &{}\quad \ldots &{}\quad ({\tilde{\upsilon }_{1n}^L}, {\tilde{\upsilon }_{1n}^U})\\ ({\tilde{\upsilon }_{21}^L}, {\tilde{\upsilon }_{21}^U}) &{}\quad 0 &{}\quad ({\tilde{\upsilon }_{23}^L}, {\tilde{\upsilon }_{23}^U}) &{}\quad \ldots &{} \quad ({\tilde{\upsilon }_{2n}^L}, {\tilde{\upsilon }_{2n}^U})\\ \vdots &{}\quad \vdots &{}\quad \vdots &{}\quad \ddots &{}\quad \vdots \\ ({\tilde{\upsilon }_{n1}^L}, {\tilde{\upsilon }_{n1}^U}) &{}\quad ({\tilde{\upsilon }_{n2}^L}, {\tilde{\upsilon }_{n2}^U})&{}\quad ({\tilde{\upsilon }_{n3}^L}, {\tilde{\upsilon }_{n3}^U}) &{}\quad \ldots &{}\quad 0 \end{bmatrix}.\end{aligned}$$

**Step 5:**

The NWIVHF aggregated elements are given as follows:

$$\begin{aligned}F=\begin{bmatrix} 0 &{}\quad \Phi _{12}&{}\quad \Phi _{13} &{}\quad \ldots &{}\quad \Phi _{1n}\\ \Phi _{21} &{}\quad 0 &{}\quad \Phi _{23} &{}\quad \ldots &{}\quad \Phi _{2n}\\ \vdots &{}\quad \vdots &{}\quad \vdots &{}\quad \ddots &{}\quad \vdots \\ \Phi _{n1} &{}\quad \Phi _{n2}&{}\quad \Phi _{n3} &{}\quad \ldots &{} \quad 0 \end{bmatrix}.\end{aligned}$$

The aggregated formula is

11 $$\begin{aligned} \Phi _{ij}=\frac{1}{\#\tilde{\upsilon }^L}(\tilde{\upsilon }_{ij}^L)^2-\frac{1}{\#\tilde{\upsilon }^U}(\tilde{\upsilon }_{ij}^U)^2. \end{aligned}$$

The construction of the normalized decision matrix *Q* can be described as follows:

12 $$\begin{aligned} \Phi _{ij}=\frac{\Phi }{l}. \end{aligned}$$

Here,

13 $$\begin{aligned}{} & {} =max\left\{ max \sum _{j=1}^{n} \Phi _{ij},\ max \sum _{i=1}^{n} \Phi _{ij}\right\} , \ \ \ i, j \in 1,2,...,n. \nonumber \\{} & {} Q=\begin{bmatrix} 0 &{}\quad \tilde{\Phi }_{12}&{}\quad \tilde{\Phi }_{13} &{}\quad \ldots &{}\quad \tilde{\Phi }_{1n}\\ \tilde{\Phi }_{21} &{}\quad 0 &{}\quad \tilde{\Phi }_{23} &{}\quad \ldots &{} \quad \tilde{\Phi }_{2n}\\ \vdots &{}\quad \vdots &{}\quad \vdots &{}\quad \ddots &{}\quad \vdots \\ \tilde{\Phi }_{n1} &{}\quad \tilde{\Phi }_{n2}&{}\quad \tilde{\Phi }_{n3} &{}\quad \ldots &{}\quad 0 \end{bmatrix}. \end{aligned}$$

**Step 6:**

Determine the total-influence matrix *K*, which depends on the following equation:

14 $$\begin{aligned} K=Q(I-Q)^{-1}. \end{aligned}$$

Here, the identity matrix is denoted by *I*.

**Step 7:**

Construct *d* and *r* which are the influence vectors. *d* denotes the influence vector and *j* is the influence factor, Here, $(j=1,2,\dots ,n)$. Then, the sum of the row values in the total-influence matrix is defined as *d*. Further, *r* denotes the influence vector and *i* is the influence factor, where $(i= 1,2,\dots ,n)$. Then, the sum of each column value in the total-influence matrix is defined as *r*. The plotting set $\{((d_{i}+r_{i}), (d_{i}-r_{i}))\i =1,2,...,n\}$ helps to construct the cause and effect graph. Here, $i=j$, and finally, $(d_{i}-r_{i})$ value represents the effect group. $(d_{i}+r_{i})$ values represent the total importance.

**Step 8:** The weight value of each criteria are calculated by the following equation:

15 $$\begin{aligned} w_{i}=[(d_{i}+ r_{i})^2+(d_{i}- r_{i})^2]. \end{aligned}$$

Finally, the weight of the normalized criteria is described as follows:

16 $$\begin{aligned} W_{i}=\frac{w_{i}}{\sum _{i=1}^{n}w_{i}}, \end{aligned}$$

where criteria weight is represented as $W_{i}$.

### Proposed normal wiggly interval-valued hesitant fuzzy PROMETHEE-II method

In this subsection, we proposed normal wiggly interval-valued hesitant fuzzy PROMETHEE-II method. This set is an extension of the hesitant fuzzy set.

Then, we consider the alternative $$ A_{i}(i=1,2,3,...,m)$$ based on their selected criteria $$ C_{j}(j=1,2,3,...,n)$$. Let NWIVHFE be as follows:

17 $$\begin{aligned} H_{NWIVHFS}=\left\{ \left\langle \tilde{\upsilon }_{\tilde{U}}(u_{i}), \tilde{\alpha }(\tilde{\upsilon }_{\tilde{U}}(u_{i}^{L}), \tilde{\upsilon }_{\tilde{U}}(u_{i}^{U}))\right\rangle \in U\right\} .\nonumber \\ \end{aligned}$$

**Step 1:** Calculate the NWIVHFDM values as shown in Table 2.

**Step 2:** The following equation is used to determine the score function. Then, $\left\langle \tilde{\upsilon }, \alpha (\tilde{\upsilon }) \right\rangle$ is the score function.

18 $$\begin{aligned}{} & {} S_{NWIVHPFS}\nonumber \\{} & {} =\frac{1}{2}\Big [\Big ( \mu (\bar{\upsilon }^{L}{-} \sigma _{\upsilon }^{L}) {+} (1-\mu ) \Big (\frac{1}{\# \upsilon } \sum _{i=1}^{\# \upsilon } \tilde{\bar{\upsilon _i}}^{L}-\sigma _{\tilde{\delta _i}}^{L}\Big ) \Big )^{2} \nonumber \\{} & {} +\Big ( \mu (\bar{\upsilon }^{U}- \sigma _{\delta }^{U}) + (1-\mu )\nonumber \\{} & {} \times \Big ( \frac{1}{\# \upsilon } \sum _{i=1}^{\# \upsilon } \tilde{\bar{\upsilon _i}}^{U} -\sigma _{\tilde{\delta _i}}^{U}\Big ) \Big )^{2} \Big ]. \end{aligned}$$

**Step 3:**

Construct the value differences of the $i ^ {th}$ alternatives, though there are other alternatives. The differences in quantitative values between the alternatives are calculated.

**Step 4:**

Determine the preference function $P_j (i, i ')$. The PROMETHEE model recommends six different types of options. This option function is preferred for its consideration of priority criteria, such as preferences and levels of negligence. In real-time applications, it is difficult for the determiner to determine which option function is appropriate for each criterion and to measure the relevant criteria. The simplified preference function is defined as follows:

19 $$\begin{aligned}{} & {} Z_{j}(i,i') = 0 if \;\;\; Z_{ij} \le Z_{i'j}, \end{aligned}$$

20 $$\begin{aligned}{} & {} Z_{j}(i,i') = (Z_{ij} - Z_{i'j})\;\;\;\; if\;\; Z_{ij} \ge Z_{i'j}. \end{aligned}$$

**Step 5:**

The weighted criteria are calculated based on the NWIVHF-DEMATEL method. This method is one of the most promising weight finding solutions. The weight value is calculated based on Normal wiggly interval-valued hesitant fuzzy set.

**Step 6:**

Construct the aggregated preference function of each of the criteria weights. The aggregated preference function formula is given as:

21 $$\begin{aligned} \Delta (i,i')=\Bigg [\sum _{j=1}^{m} W_j.Z_j(i,i')\Bigg ]/\sum _{j=1}^{m} W_{j}, \end{aligned}$$

where $W_j$ represents the relative importance of the criterion weight. **Step 7:**

Determine the positive and negative flows of the alternatives. The determination of the positive and negative flows is as given below:

22 $$\begin{aligned}{} & {} \Phi ^+(i)=1/n-1 \sum _{i=1}^{n} \Delta (i,i') \end{aligned}$$

23 $$\begin{aligned}{} & {} \Phi ^-(i)=1/n-1 \sum _{i=1}^{m} \Delta (i,i^{\prime }). \end{aligned}$$

Other names for positive and negative flows are leaving and entering flows. Here, *n* represents the alternative and $(n-1)$ represents the number of other alternatives.

**Step 8:**

Calculate the net outranking flow $\Phi (i)$ to deliver the overall preference degrees of the alternatives *i* and $i^{\prime }$.

24 $$\begin{aligned} \Phi (i) = \Phi ^+(i)- \Phi ^-(i). \end{aligned}$$

**Step 9:**

Rank the alternative based on the outranking flow. The best alternative is the highest value of outranking flow $\Phi (i)$.

## Illustrative example

In this part, we determine the bond between each criterion. In Normal wiggly interval-valued hesitant fuzzy set, we discuss our proposed method NWIVHF-DEMATEL with one of the unique effective weight-finding method and different procedure of MCDM weight-finding method, the DEMATEL method, is used as the weight detection method. Furthermore, The selection of alternatives is chosen by PROMETHEE-II method. Different types of bioenergy production modes are chosen as alternatives:

- **Gasification **($$A_1$$)
- **Biodiesel **($$A_2$$)
- **Bioethanol **($$A_3$$)
- **Biogas **($$A_4$$).

These four different types of alternatives are determined based on the following criteria:

- **Fuel cost **($$C_1$$)
- **Technical cost **($$C_2$$)
- **Environmental safety **($$C_3$$)
- $$CO_2$$ **emission level** ($$C_4$$).

Each value of decision matrix is based on the decision maker’s hesitancy. The decision makers give the values of their own analysis of each criteria. The matrix values are placed in ascending order. Each value of criteria is independent. Decision makers analyze the alternative and give their opinion based on the criteria.

Now, we determine the causal relationships between the criteria. For that, Fig. 2 linguistic scale values are used to determine the *D* direct-influence matrix. Equation (4) is used to develop the matrix. Table 3 represents the direct-influence matrix values.

**Table 3:** Decision matrix with NWHPFs

	$C_{1}$	$C_{2}$	$\cdots$	$C_{n}$
$A_{1}$	$\big \langle \upsilon _{11},\alpha (\upsilon ^{L}_{11}, \upsilon ^{U}_{11})\big \rangle$	$\big \langle \upsilon _{12},\alpha (\upsilon ^{L}_{12}, \upsilon ^{U}_{12})\big \rangle$	$\cdots$	$\big \langle \upsilon _{1n},\alpha (\upsilon ^{L}_{1n}, \upsilon ^{U}_{1n})\big \rangle$
$A_{2}$	$\big \langle \upsilon _{21},\alpha (\upsilon ^{L}_{21}, \upsilon ^{U}_{21})\big \rangle$	$\big \langle \upsilon _{22},\alpha (\upsilon ^{L}_{22}, \upsilon ^{U}_{22})\big \rangle$	$\cdots$	$\big \langle \upsilon _{2n},\alpha (\upsilon ^{L}_{2n}, \upsilon ^{U}_{2n})\big \rangle$
$\vdots$	$\vdots$	$\vdots$	$\vdots$	$\vdots$
$A_{m}$	$\big \langle \upsilon _{m1},\alpha (\upsilon ^{L}_{m1}, \upsilon ^{U}_{m1})\big \rangle$	$\big \langle \upsilon _{m2},\alpha (\upsilon ^{L}_{m2}, \upsilon ^{U}_{m2})\big \rangle$	$\cdots$	$\big \langle \upsilon _{mn},\alpha (\upsilon ^{L}_{mn}, \upsilon ^{U}_{mn})\big \rangle$

**Table 4:** Direct-influence matrix *D*

	$C_1$	$C_2$	$C_3$	$C_4$
$C_1$	$\{[0.5, 0.5], [0.5, 0.5],$ $[0.5, 0.5]\}$	$\{[0.85, 0.15], [0.9, 0.1]$ $[0.75, 0.25]\}$	$\{[0.65, 0.35], [0.75, 0.25]$, $[0.45, 0.55]\}$	$\{[0.75, 0.25], [0.85, 0.15]$ $[0.65, 0.35]\}$
$C_2$	$\{[0.65, 0.35], [0.5,$ $0.5], [0.75, 0.25]\}$	$\{[0.5, 0.5], [0.5, 0.5],$ $[0.5, 0.5]\}$	$\{[0.15, 0.75], [0.35,$ $0.65], [0.5, 0.5]\}$	$\{[0.15, 0.85], [0.25,$ $0.75], [0.35, 0.65]\}$
$C_3$	$\{[0.45, 0.55], [0.5,$ $0.5], [0.5, 0.5]\}$	$\{[0.15, 0.85], [0.25,$ $0.75], [0.25, 0.75]\}$	$\{[0.5, 0.5], [0.5,$ $0.5], [0.5, 0.5]\}$	$\{[0.35, 0.65], [0.25,$ $0.75], [0.15, 0.85]\}$
$C_4$	$\{[0.25, 0.75], [0.5,$ $0.5], [0.45, 0.55]\}$	$\{[0.35, 0.65], [0.15,$ $0.85], [0.15, 0.85]\}$	$\{[0.15, 0.85], [0.35,$ $0.65], [0.25, 0.75]\}$	$\{[0.5, 0.5], [0.5,$ $0.5], [0.5, 0.5]\}$

The pairwise comparison of bonding depends upon each criterion. This bonding is developed by the decision makers. Equations (9) and (10) are used to calculate the normalized matrix. $\zeta =0.5$ is the risk preference value. The decision maker’s risk preference value is evaluated. The direct-influence matrix is determined by using Step (4).

**Fig. 3:**
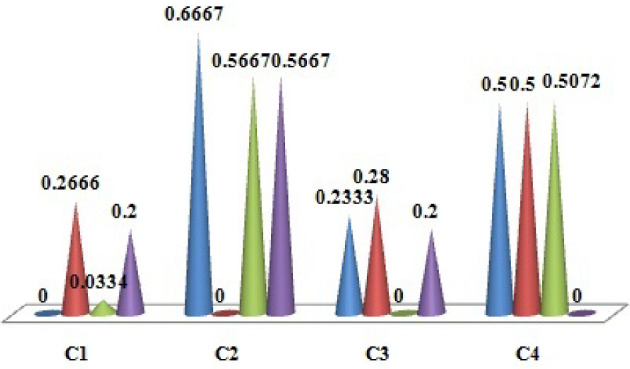
Values of single-valued direct-influence matrix

Equation (11) is used to evaluate the single-valued direct-influence matrix. Each value of the direct-influence matrix is aggregated. By aggregation, the value of the direct-influence matrix is used to get the single-valued direct-influence matrix. Table 4 represents the matrix value and Fig. 3 shows the single-valued direct-influence matrix result.

**Fig. 4:**
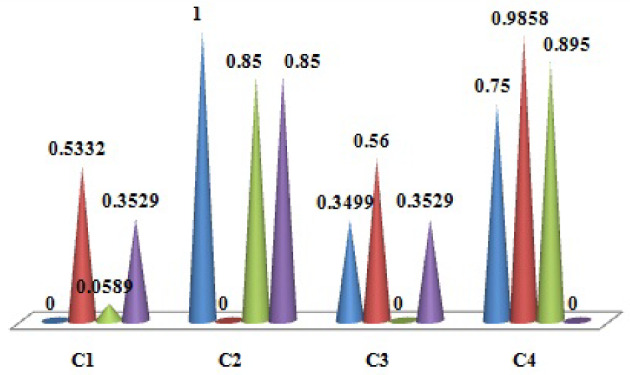
Values of normalized direct-influence matrix

Finally, Eqs. (12) and (13) are used to evaluate the normalized direct-influence matrix *Q*. Table 5 represents the value of *Q*. Figure 4 shows the values of the normalized direct-influence matrix.

Then, Eq. (14) is used to evaluate the total-influence matrix *K*. Table 6 represents the total-influence matrix value.

According to Step 7 of the algorithm, the influence vectors *d* and *r* are evaluated. Table 7 represents the $d_i$ and $r_i$ values.

**Fig. 5:**
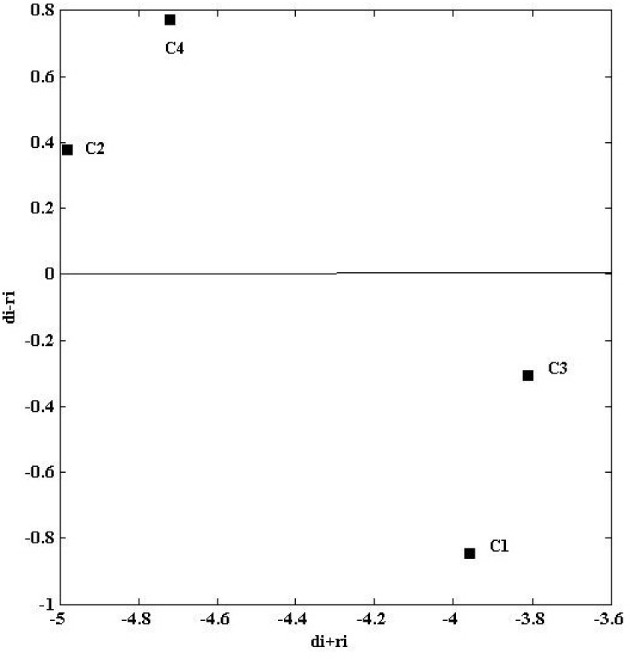
Cause and effect graph results

We obtain the cause and effect graph from the $d_i$ & $r_i$ values, and Fig. 5 plots the cause and effect graph results.

**Fig. 6:**
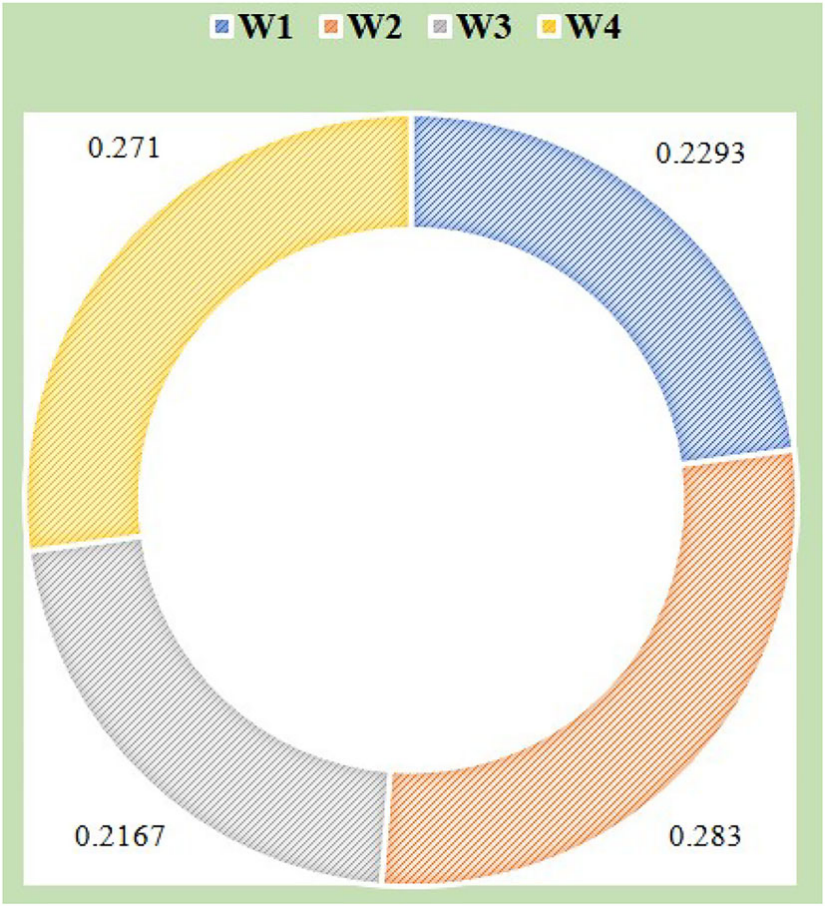
Values of weighted criteria

Equations (15) and (16) are used to evaluate the criteria weights. Figure 6 shows the normalized criteria weight results.

**Table 5:** Single-valued direct-influence matrix *F*

	$C_1$	$C_2$	$C_3$	$C_4$
$C_1$	0	0.6667	0.2333	0.5
$C_2$	0.2666	0	0.28	0.5
$C_3$	0.0334	0.5667	0	0.5072
$C_4$	0.2	0.5667	0.2	0

**Table 6:** Normalized direct-influence matrix *Q*

	$C_1$	$C_2$	$C_3$	$C_4$
$C_1$	0	1	0.3499	0.7500
$C_2$	0.5332	0	0.5600	0.9858
$C_3$	0.0589	0.8500	0	0.8950
$C_4$	0.3529	0.8500	0.3529	0

**Table 7:** Total-influence matrix *K*

	$C_1$	$C_2$	$C_3$	$C_4$
$C_1$	$-$0.6080	$-$0.6121	$-$0.4601	$-$0.7212
$C_2$	$-$0.2735	$-$1.0791	$-$0.3507	$-$0.5970
$C_3$	$-$0.4293	$-$0.5216	$-$0.6161	$-$0.4925
$C_4$	$-$0.2457	$-$0.4673	$-$0.3250	$-$0.9358

**Table 8:** $d_{i}$ and $r_{i}$ values

	$d_{i}$	$r_{i}$	$d_{i}+r_{i}$	$d_{i}-r_{i}$	$w_{i}$	*W*
$C_1$	$-$2.4014	$-$1.5565	$-$3.9579	$-$0.8449	4.0471	0.2293
$C_2$	$-$2.3003	$-$2.6801	$-$4.9804	0.3798	4.9949	0.2830
$C_3$	$-$2.0595	$-$1.7519	$-$3.8114	$-$0.3076	3.8238	0.2167
$C_4$	$-$1.9738	$-$2.7465	$-$4.7203	0.7727	4.7831	0.2710

**Table 9:** Normal wiggly interval-valued hesitant fuzzy decision making

	$C_1$	$C_2$	$C_3$	$C_4$
$A_1$	$\{[0.2, 0.3],[0.5, 0.6]$, $[0.6, 0.7]\}$	$\{[0.1, 0.4],[0.3, 0.6]$, $[0.2, 0.3]\}$	$\{[0.1, 0.2],[0.2, 0.4]$, $[0.3, 0.5]\}$	$\{[0.2, 0.3],[0.5, 0.7]$, $[0.5, 0.7]\}$
$A_2$	$\{[0.1, 0.5],[0.3, 0.4]$, $[0.5, 0.6]\}$	$\{[0.5, 0.7],[0.3, 0.5]$, $[0.2, 0.5]\}$	$\{[0.2, 0.3],[0.3, 0.4]$, $[0.4, 0.6]\}$	$\{[0.3, 0.7],[0.4, 0.8]$, $[0.5, 0.7]\}$
$A_3$	$\{[0.2, 0.4],[0.3, 0.6]$, $[0.5, 0.7]\}$	$\{[0.3, 0.5],[0.4, 0.7]$, $[0.6, 0.7]\}$	$\{[0.5, 0.6],[0.3, 0.5]$, $[0.4, 0.8]\}$	$\{[0.45, 0.65]$,[0.65, 0.8], $[0.5, 0.7]\}$
$A_4$	$\{[0.35, 0.4],[0.15, 0.4]$, $[0.2, 0.5]\}$	$\{[0.2, 0.5],[0.4, 0.8]$, $[0.4, 0.6]\}$	$\{[0.2, 0.2],[0.4, 0.6]$, $[0.3, 0.5]\}$	$\{[0.2, 0.4],[0.3, 0.7]$, $[0.2, 0.5]\}$

Next, we consrtuct the decision matrix values. The decision matrix values are related to the selected application. The NWIVHF decision matrix is deliver the deep and digger information of decision makers hesitation thoughts. The decision matrix is used to get the NWIVHF matrix. Both the upper and lower limit values depend on the interval-valued hesitant fuzzy set. Table 8 represents the NWIVHFDM values. Equation (17) is used to evaluate the NWIVHF matrix value.

**Fig. 7:**
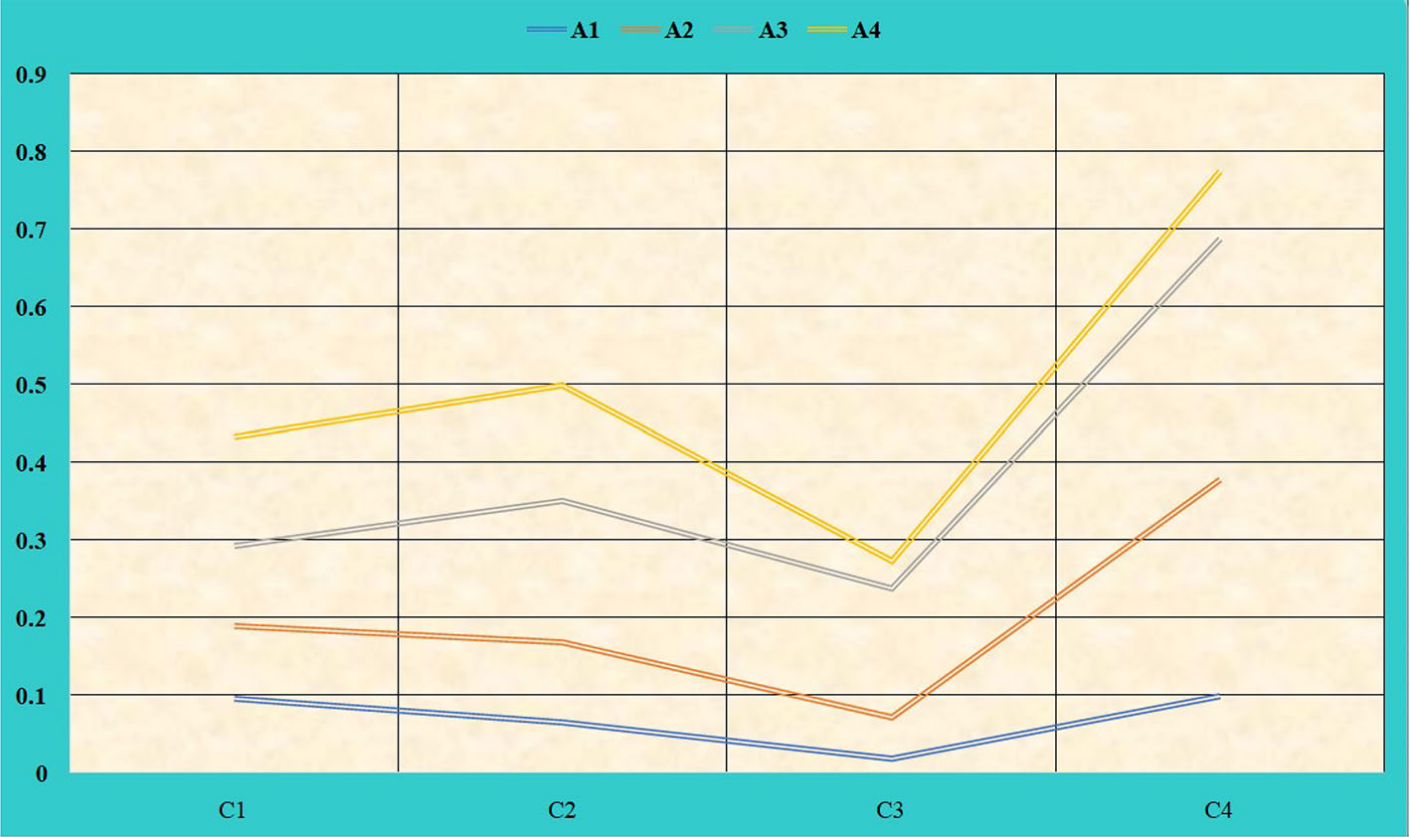
NWIVHF score matrix values

Equation (18) is used to calculate the NWIVHF score matrix. Table 9 represents the values of the score matrix, and Fig. 7 shows the score value of NWIVHF.

The differences in the quantitative values are calculated between the different alternatives. The values are given in Table 10.

The weighted values are calculated according to the NWIVHF-DEMATEL method. The preference function can be calculated using Eq. (19). The aggregated preference function is calculated based on equation (21). The values of the aggregated preference function are given in Table 11.

**Fig. 8:**
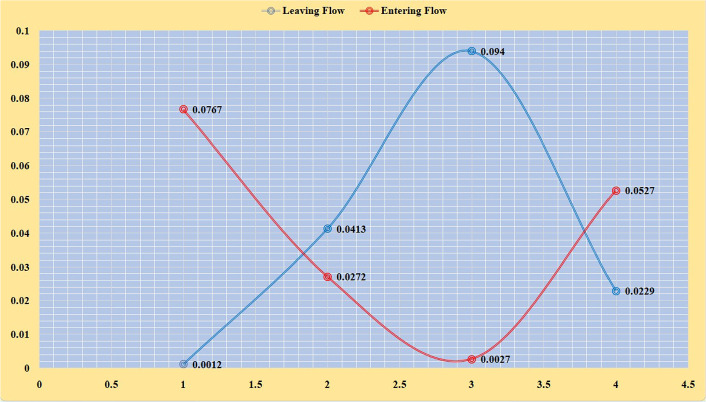
NWIVHF leaving and entering flows values

The leaving and entering flows can be calculated using equations (22) and (23). The values of the leaving and entering flow are given in Table 12 and plotted in Fig. 8.

**Fig. 9:**
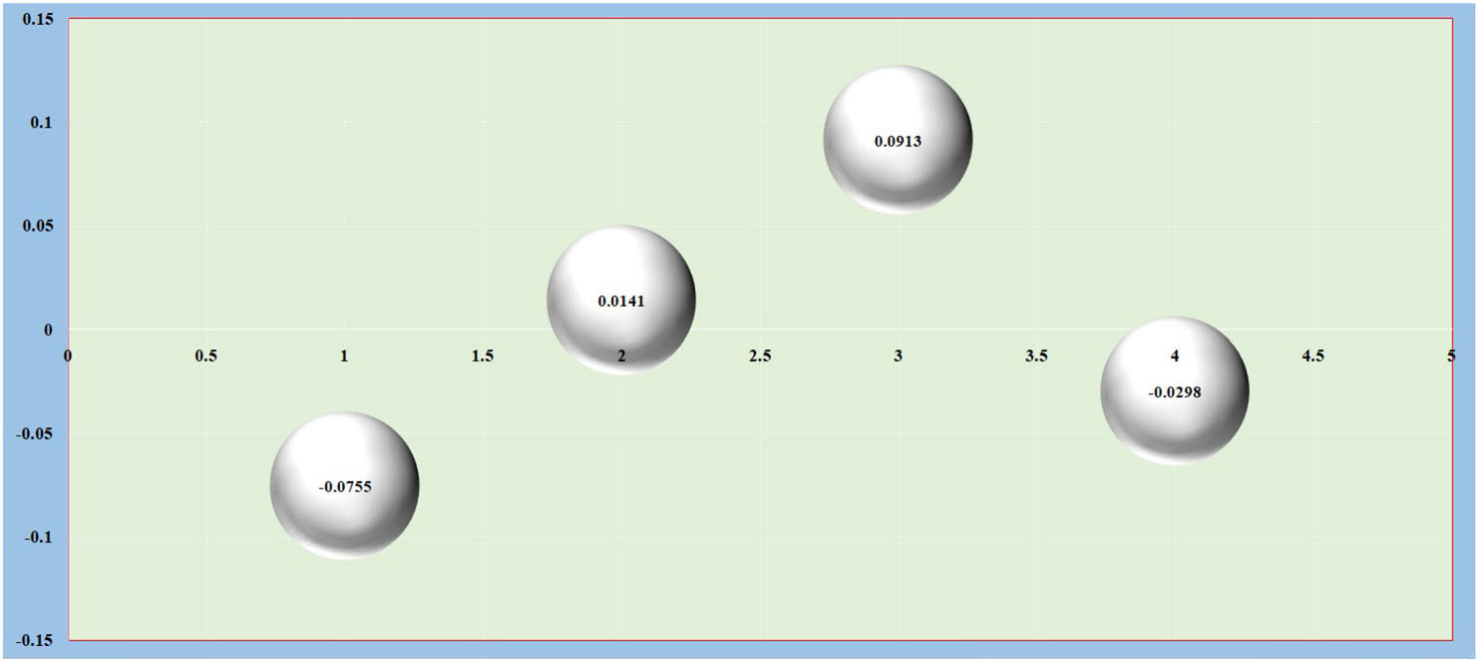
NWIVHF net flow values

The values of NWIVHF net flow are given in Table 13 and plotted in Fig. 9. Based on the net flow values, the best alternative is $A_3$ i.e., bioethanol was the most successful of the biomass production options evaluated, followed by biodiesel. Sugars, starches, lignocellulosic biomass, and algae are used to produce bioethanol [44]. Bioethanol is used as a petrol alternative in automobile engines. As a fuel, it has substantial economic and environmental advantages over petrol. Biodiesel is made from animal and vegetable oils, including used cooking oil (UCO), and may be used to power diesel engines [33]. Biogas is made up of methane and carbon dioxide, as well as trace quantities of hydrogen sulfide, mercaptans, ethane, and other pollutants [9]. Anaerobic digestion biogas may be utilized to create heat and electricity with minimal post-processing cleaning. Carbonaceous components such as hydrogen, carbon monoxide, carbon dioxide, methane, higher hydrocarbons, and nitrogen are transformed into syngas with the aid of a gasification agent and a catalyst [21]. It is one of the most efficient bio-hydrogen production methods. So far, each method has its own advantages, and the optimal use of a technology is determined by the availability of feedstock, economic resources, toxic gas emissions, and imposed regulations, waste pre-treatment process, purification of the biofuels produced, plant equipment setup, and reactor operation and maintenance. As a result, studies focusing on improving process efficacy will help in attaining sustainable biomass developments in the coming years.

**Table 10:** NWIVHF score matrix

	$C_1$	$C_2$	$C_3$	$C_4$
$A_1$	0.0951	0.0641	0.0187	0.0980
$A_2$	0.0931	0.1041	0.0518	0.2797
$A_3$	0.1044	0.1818	0.1675	0.3099
$A_4$	0.1399	0.1480	0.0348	0.0864

**Table 11:** Differences in the criteria value of the NWIVHF matrix for each alternative

	$C_1$	$C_2$	$C_3$	$C_4$
$(A_1, A_2 )$	0.002	0	0	0
$(A_1, A_3 )$	0	0	0	0
$(A_1, A_4 )$	0	0	0	0.0116
$(A_2, A_1 )$	0	0.0400	0.0331	0.1817
$(A_2, A_3 )$	0	0	0	0
$(A_2, A_4 )$	0	0	0.0170	0.1933
$(A_3, A_1 )$	0.0093	0.1177	0.1488	0.2119
$(A_3, A_2 )$	0.0113	0.0777	0.1157	0.0302
$(A_3, A_4 )$	0	0.0338	0.1327	0.2235
$(A_4, A_1 )$	0.0448	0.0839	0.0161	0
$(A_4, A_2 )$	0.0468	0.0439	0	0
$(A_4, A_3 )$	0.0355	0	0	0

**Table 12:** NWIVHF aggregated preference function values

	$C_1$	$C_2$	$C_3$	$C_4$
$A_1$	–	0.0005	0	0.0031
$A_2$	0.0677	–	0	0.0561
$A_3$	0.1250	0.0579	–	0.0990
$A_4$	0.0375	0.0231	0.0081	–

## Comparative analysis

In this section, we analyze the superiority of the proposed method by comparing the acquired results with existing fuzzy MCDM methods. Hesitant fuzzy set seeks to rectify the hesitant position of the decision maker and come up with a better solution. Its extension, the normal wiggly hesitant fuzzy set, provides an excellent solution to the deep and confusing hesitations of the decision maker. Specifically, the normal wiggly interval-valued hesitant fuzzy set provides value not only for membership, but also for non-membership functions. Also, since the normal wiggly hesitant fuzzy set is based on the triangular fuzzy set, the interval-valued hesitant fuzzy set provides the upper limit and lower limit values for membership and non-membership as well as the triangular fuzzy set.

**Fig. 10:**
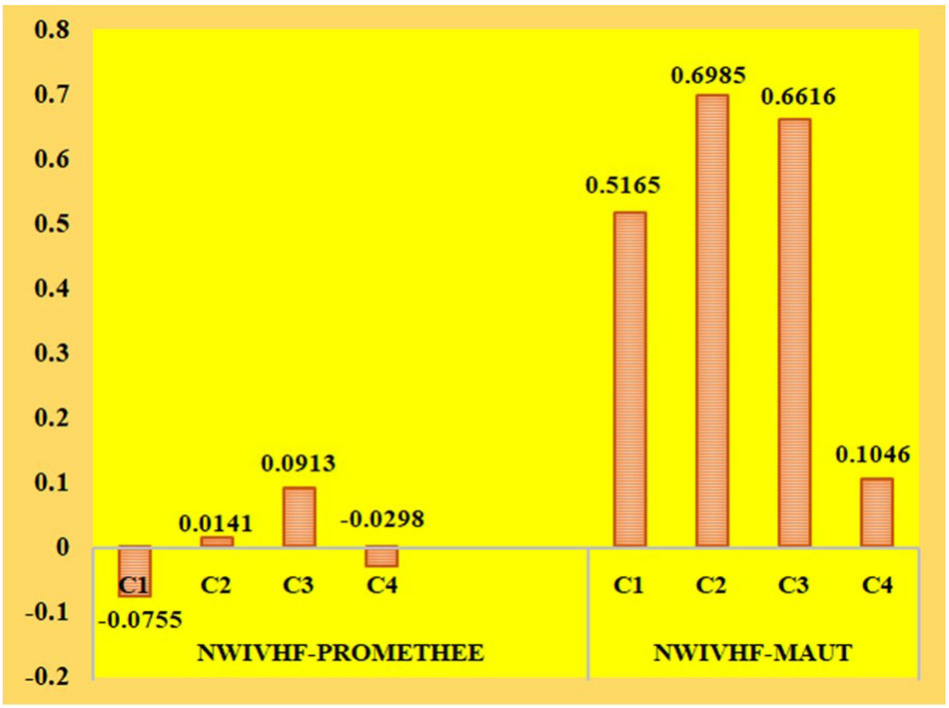
Comparison result of NWIVHF-DEMATEL and NWIVHF-CRITIC

The MCDM methods have different types of ranking and weight detection methods. Here, we analyzed the alternative changes of our obtained results from proposed methods with two prominent weight detection and ranking methods. Here, CRITIC is the weight detection method which is compared with the DEMATEL method and MAUT is the ranking method which is compared by the PROMETHEE method. CRITIC method does not take into account the type of criteria, whereas DEMATEL is a robust approach to causal analysis that allows researchers to split the involved criteria of a system into cause and effect groups, which has assisted decision makers in recognizing the criteria that hold the greatest influence. The subjective evaluation of criteria weights using the DEMATEL technique has given precedence to the technical cost and $CO_2$ emission levels, but the objective evaluation has given preference to the fuel cost and environmental safety, as shown in Table 15 and its graphical depiction in Fig. 10. Weighting methods have also played an important role in establishing the possible technology for biomass processing.

**Table 13:** NWIVHF leaving and entering flow values

	Leaving flow	Entering flow
$A_1$	0.0012	0.0767
$A_2$	0.0413	0.0272
$A_3$	0.0940	0.0027
$A_4$	0.0229	0.0527

**Table 14:** NWIVHF net flow values

	Net flow	Rank
$A_1$	$-$0.0755	4
$A_2$	0.0141	2
$A_3$	0.0913	1
$A_4$	$-$0.0298	3

**Fig. 11:**
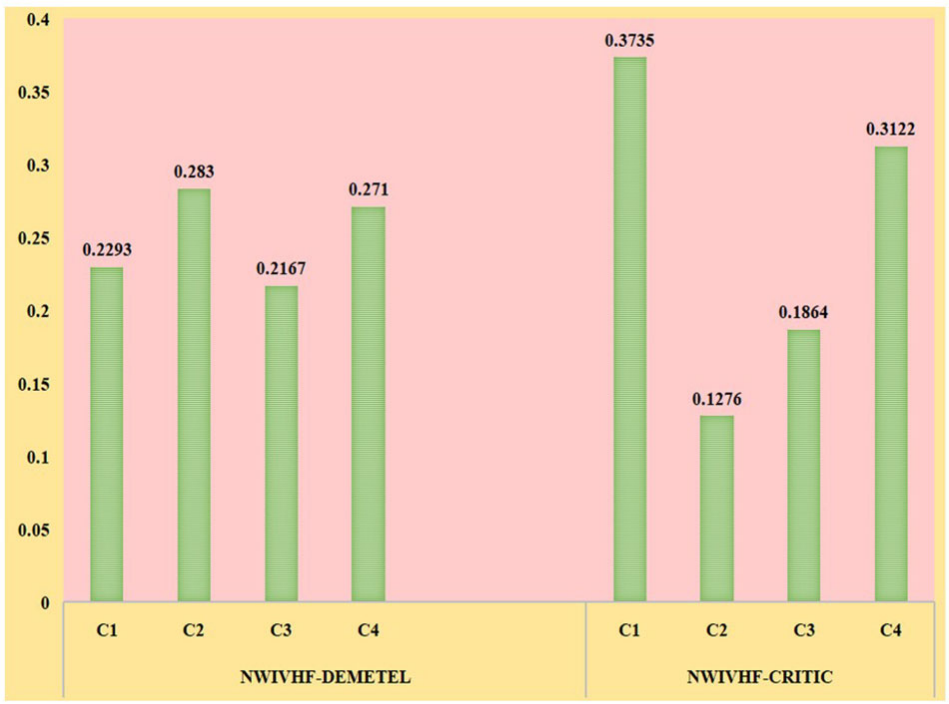
Comparison result of NWIVHF-PROMETHEE and NWIVHF-MAUT

The precise derivation and depiction of the DM’s preference pose challenges in MAUT. By evaluating the thresholds of indifference and stringent preference, PROMETHEE makes it straightforward to investigate DM’s sense. The preferences of the alternatives, on the other hand, are obtained by comparing them pairwise, making it superior to the considered approach. Table 16 and Fig. 11 show that the utility-based approach and the preference-based approach both recommended distinct methods for commercializing biomass. The analyzed MCDM approaches recommend biodiesel and bioethanol as viable biomass technologies. Yet, due to its high octane rating, excellent combustion efficiency, and environmental safety, bioethanol may be considered a potential alternative to biodiesel. According to the study, the use of an appropriate decision-making approach will assist in the development of a workable solution to the problem. Furthermore, using a hybrid weighting approach to determine the relevance of the criterion will help to provide robust findings for the problem.

## Conclusion

The rising advancements in renewable sources show the potential capabilities of biomass energy in tackling the problem of energy demand and making it a future-proof energy source. Unlike other renewable energy sources, biomass energy might be used to generate electricity and develop alternative transportation fuels. Due to its ability to lower carbon emissions, bioenergy has the potential to replace energy produced through the combustion of fossil fuels. However, the intricacy of selecting an appropriate bioenergy production mode based on biomass necessitates the careful consideration of multiple elements, which has been effectively handled utilizing the fuzzy MCDM technique. The NWIVHFS effectively manages the ambiguous data supplied by the experts for the topic under consideration, assisting in obtaining trustworthy results. The DEMATEL method, in conjunction with PROMETHEE-II, which has been enhanced utilizing the NWIVHFS, contributes to the best bioenergy production technology. Decision makers are able to understand the complexity of a situation and make informed decisions, since the DEMATEL method deals with the interdependent relationships between the chosen criteria. Some of the method’s strengths include figuring out the causal connections between evaluation criteria and successfully avoiding imprecise and vague assessments. A workable solution to the problem under consideration has been produced as a result of using the

**Table 15:** Comparison result of NWIVHF-DEMATEL and NWIVHF-CRITIC

Methods	$C_{1}$	$C_{2}$	$C_{3}$	$C_{4}$
NWIVHF-DEMATEL	0.2293	0.2830	0.2167	0.2710
NWIVHF-CRITIC	0.3735	0.1276	0.1864	0.3122

PROMETHEE-II approach to identify the practical form of bioenergy production. Prioritizing alternatives by pairwise comparison is a unique characteristic of this technique. The method’s qualities include clarity, stability, and the capacity to handle complete and partial data at the same time. The complexity of the procedure, on the other hand, could be a limitation of the PROMETHEE-II method. Due to its high-octane rating and outstanding combustion efficiency, bioethanol stands out among the alternatives as a viable method of producing bioenergy; as a result, it is considered to be environmentally beneficial. When compared to other existing methods, the findings of the comparative analysis demonstrate that the created methodology is workable for managing practical challenges. This demonstrates how the established framework is preferable to alternative approaches. A potential future extension of the problem would be to analyze the various feedstock for the manufacture of bioethanol and expand the effort to include group decision making with the help of extended NWHFS.

**Table 16:** Comparison result of NWIVHF-PROMETHEE and NWIVHF-MAUT

Alternatives	PROMETHEE	Ranking	MAUT	Ranking
$A_{1}$	$-$0.0755	IV	0.5165	III
$A_{2}$	0.0141	II	0.6985	I
$A_{3}$	0.0913	I	0.6616	II
$A_{4}$	$-$0.0298	III	0.1046	IV
